# Sensitivity to interaural time differences and localization accuracy in cochlear implant users with combined electric-acoustic stimulation

**DOI:** 10.1371/journal.pone.0241015

**Published:** 2020-10-19

**Authors:** Monika Körtje, Uwe Baumann, Timo Stöver, Tobias Weissgerber

**Affiliations:** 1 Audiological Acoustics, ENT Department, University Hospital Frankfurt, Goethe University Frankfurt, Frankfurt am Main, Germany; 2 ENT Department, University Hospital Frankfurt, Goethe University Frankfurt, Frankfurt am Main, Germany; University of Melbourne, AUSTRALIA

## Abstract

**Objectives:**

In this study, localization accuracy and sensitivity to acoustic interaural time differences (ITDs) in subjects using cochlear implants with combined electric-acoustic stimulation (EAS) were assessed and compared with the results of a normal hearing control group.

**Methods:**

Eight CI users with EAS (2 bilaterally implanted, 6 unilaterally implanted) and symmetric binaural acoustic hearing and 24 normal hearing subjects participated in the study. The first experiment determined mean localization error (MLE) for different angles of sound incidence between ± 60° (frontal and dorsal presentation). The stimuli were either low-pass, high-pass or broadband noise bursts. In a second experiment, just noticeable differences (JND) of ITDs were measured for pure tones of 125 Hz, 250 Hz and 500 Hz (headphone presentation).

**Results:**

Experiment 1: MLE of EAS subjects was 8.5°, 14.3° and 14.7°, (low-, high-pass and broadband stimuli respectively). In the control group, MLE was 1.8° (broadband stimuli). In the differentiation between sound incidence from front and back, EAS subjects performed on chance level. Experiment 2: The JND-ITDs were 88.7 μs for 125 Hz, 48.8 μs for 250 Hz and 52.9 μs for 500 Hz (EAS subjects). Compared to the control group, JND-ITD for 125 Hz was on the same level of performance. No statistically significant correlation was found between MLE and JND-ITD in the EAS cohort.

**Conclusions:**

Near to normal ITD sensitivity in the lower frequency acoustic hearing was demonstrated in a cohort of EAS users. However, in an acoustic localization task, the majority of the subjects did not reached the level of accuracy of normal hearing. Presumably, signal processing time delay differences between devices used on both sides are deteriorating the transfer of precise binaural timing cues.

## Introduction

Accurate localization of sound incidence is of utmost importance to prevent potential dangerous situations in everyday life (e.g. traffic situations). Furthermore, acoustic localization supports auditory scene analysis and improves speech perception e.g. in noisy environments. Interaural time differences (ITDs) and interaural level differences (ILDs) are the most relevant cues for the localization of sound sources. Normal hearing persons (NH) are most sensitive for ILD cues in higher frequencies above 1.5 kHz, whereas ITDs are relevant for sound localization of signals with lower frequencies, explored by e.g. Blauert in 1997 [1]. Additionally, ITD cues derived by analysis of the envelope of high frequency sounds are also involved in localization, but with less relevance [2, 3]. Unless otherwise stated below, the term “ITD sensitivity” refers to the sensitivity of a bilateral time difference caused by the temporal fine structure of a stimulus. Sensitivities of ITD and ILD are highly correlated and a complex interaction between both neuronal identification networks can be assumed, even when hearing sensitivity is deteriorated as in persons with hearing loss [4–6].

For users of cochlear implants (CIs), sound localization and speech perception in noise is still a challenging task. This applies in particular to communication situations in noisy environments. Studies have shown that some CI users are sensitive to ITD cues measured in psychoacoustic setups, such as with simultaneous CI stimulation and contralateral acoustic stimulation [7, 8]. Furthermore CI users are able to make use of binaural cues to localize sounds [3, 4, 9]. However, the level of performance reported in localization studies is way below NH [9].

One potential reason for deteriorated binaural cue sensitivity is the lack of binaural synchronization in unilateral CI users with contralateral hearing aid or normal hearing. Electric stimulation of the auditory nerve using a cochlear implant is potentially faster compared to acoustic stimulation with slower acoustic travelling wave [10]. In addition, ITD detection is affected by the processing latencies of hearing aids with delays typically measured between 3 and 11 ms [11]. In bimodal CI users (one ear CI and one ear hearing aid), the processing delay of a CI or the contralateral hearing aid is typically unknown and therefore presumably in mismatch. However, adequate compensation of processing delay is required to achieve best prerequisites for ITD processing. Matched interaural delays may lead to better speech perception and sound localization [12, 13]. Another aspect is that in the case of severe hearing loss, especially at higher frequencies, the hearing aids can only partially compensate for the lack of audibility. Insufficient high frequency audibility can therefore also affect the localization accuracy, which could be caused by missing ILD cues from the hearing aid side.

In 2014 Jones et al. hypothesized that the lack of localization performances in bilaterally implanted CI users is probably caused by CI programming strategies and is not the result of neural impairment of central areas [14]. The authors concluded that improvement of programming strategies and better understanding of human binaural information processing is of particular importance for better localization accuracies.

For patients with substantial residual hearing in the lower frequency range but poor speech perception, implantation of a CI with hearing preservation (soft surgery procedure) enables combined electric-acoustic stimulation (EAS) to provide enhanced hearing and restore speech perception [15]. The first EAS results were described by von Ilberg et al. in 1999 [16]. For over 20 years, patients with residual low-frequency acoustic hearing have been treated with EAS and surgical techniques and implants are continually improved [17]. The benefits of acoustic low-frequency hearing are often long-term preserved since residual hearing remains stable in many patients [18]. EAS improves speech perception, especially in demanding hearing situations with multiple noise sources [19–23]. While bilateral and bimodal CI users with a hearing aid in the contralateral ear mainly use ILD information to localize sounds [9, 24], if residual hearing in the implanted ear is preserved, low-frequency acoustic hearing and ITD cues seem to support the localization of sounds in EAS users [25]. However, the just noticeable difference (JND) in ITD has shown to be considerably poorer and sometimes even out of the physiological range of about 700 μs [26, 27]. It has been shown that symmetrical acoustic residual hearing is beneficial for sound localization [28].

Only a small number of studies investigated acoustic ITD sensitivity in EAS users so far [26, 29]. The impact of potentially available ITD cues on localization accuracy is still unclear. Therefore, the aim of this study was to determine localization accuracy and JND-ITD in EAS users. A cohort of EAS users with moderate to severe contralateral hearing loss as well as a normal hearing control group was recruited to investigate ITD sensitivity in the lower frequency range. In addition, the localization performance of EAS subjects was investigated with different stimuli and front/back confusion was assessed. If EAS subjects are able to make use of ITD cues, better localization accuracies for low-pass stimuli than high-pass stimuli are expected. In addition, the impact of the degree of interaural asymmetry and the mean hearing threshold of both ears in pure tone audiogram thresholds of low frequencies on JND-ITD as well as the duration of EAS experience on ITD sensitivity and localization accuracy was examined.

## Materials and methods

### Subjects

Eight CI recipients with hearing preservation in the implanted ear (mean age: 48 years, range from 31 to 55 years; 5 female and 3 male) and 24 NH (mean age: 28 years range from 21 to 43 years; 15 female and 9 male) took part in the study. The inclusion criterion for the NH group was sufficient hearing with thresholds better than 30 dB HL in all frequencies between 250 Hz and 8 kHz. Demographical data of the EAS group is shown in Table 1. Demographical data of the NH subjects is shown in S1 Table. All EAS subjects suffered from progressive sensory hearing loss prior to CI implantation. In one subject, hearing loss was caused by sudden deafness, one had a family history of hearing loss, one had a hearing impairment since birth (probably caused by postnatal respiratory disorder), and five participants have an unknown etiology. With the exception of one participant with hearing impairment since birth, all subjects had postlingual hearing loss and most of them showed stable residual hearing in the year before the study. One subject experienced a slight progression with a maximum drop in hearing threshold of 25 dB for 125 Hz. The hearing deprivation phase describes the time period of comprised hearing before CI provision. The interaural asymmetry was defined as the difference in hearing thresholds, assessed by air conduction pure tone audiometry between left and right ear.

**Table 1:** Demographic data of the EAS subjects.

subject	group	age [years]	Hearing deprivation phase [years]	EAS experience [years]	Electrode array	FMS [%]	interaural asymmetry [dB]		
							125 Hz	250 Hz	500 Hz
**P1**	BiCI	42	37	6	2x Flex24	85/ 70	0	0	0
**P2**	CI	55	34	1	Flex24	75	0	15	5
**P3**	CI	31	13	3.8	Flex20	70	10	10	20
**P4**	CI	51	13	3.6	Flex24	100	10	15	15
**P5**	CI	52	25	4.8	Flex24	70	5	15	35
**P6**	CI	53	23	3.3	Flex20	60	10	5	25
**P7**	BiCI	45	6	2.1	2x Flex24	90/ 90	5	10	20
**P8**	CI	55	1	4.8	Flex24	95	20	25	10
**mean**		**48**		**3.7**		**81**	**7.5**	**11.9**	**16.3**

FMS (Freiburger monosyllables): monosyllabic speech perception score in quiet assessed with CI EAS only (double-blocked contralateral ear). BiCI: two bilateral EAS users (two numbers at the speech perception scores are for results of the right/ left side). Interaural asymmetry: gap between pure tone audiogram thresholds of left/ right side.

**Fig 1:**
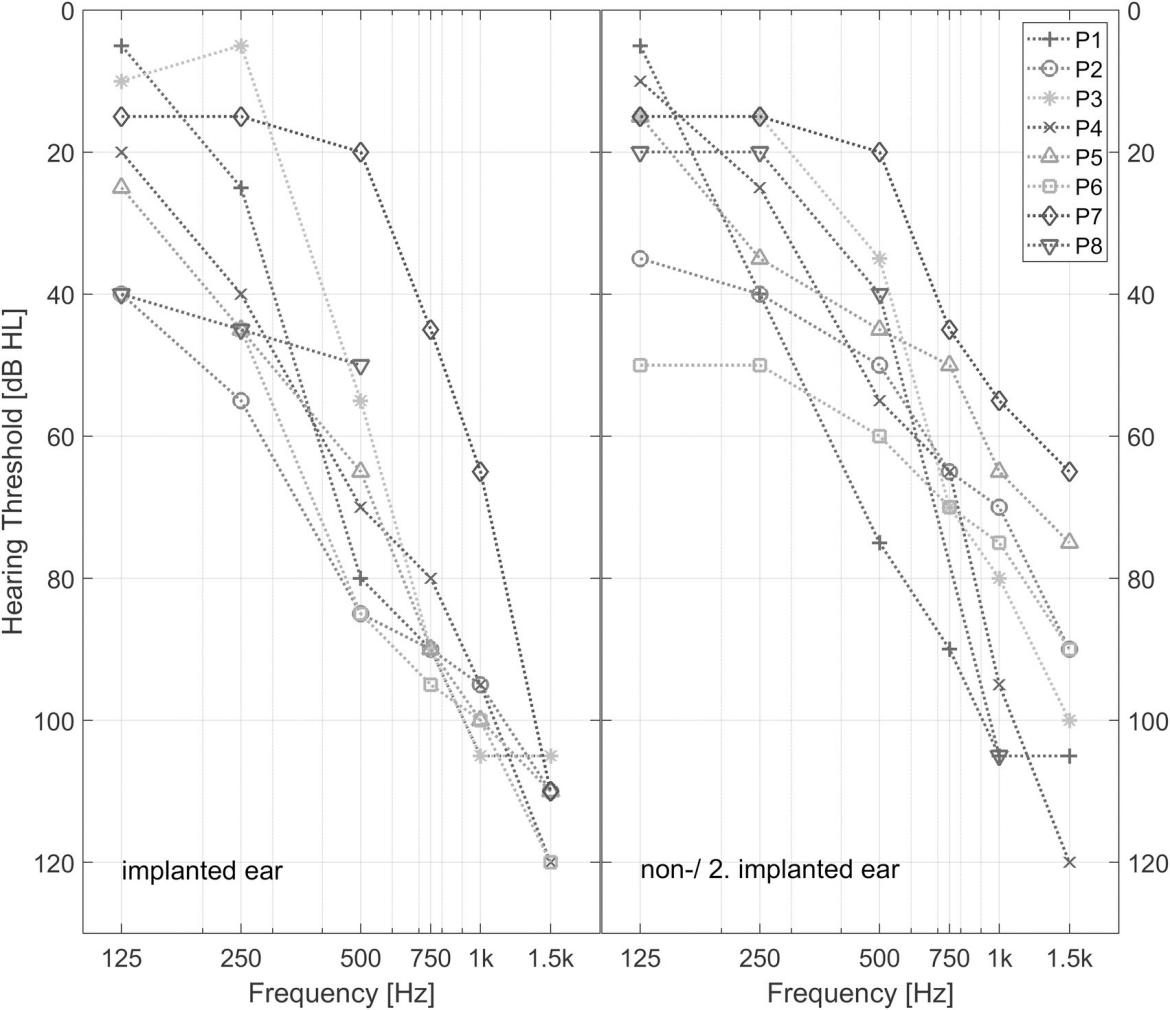
Pure tone audiogram thresholds of EAS subjects from 125 Hz to 1.5 kHz. Left: implanted side. Right: contralateral or the second implanted side. P1 and P7 are the bilateral EAS subjects. P8 had no measureable ipsilateral residual hearing for frequencies above 500 Hz.

Fig 1 shows the pure tone audiogram thresholds of the ipsi- and contralateral side (air conduction). Five participants were bimodal with a hearing aid on the contralateral side, two were bilateral EAS user with EAS on both sides and one participant was an EAS user with high frequency hearing loss on the contralateral ear without wearing a hearing aid.

MED-EL (Innsbruck, Austria) devices were used in all EAS participants. Used electrodes were Flex20 and Flex24 (see in Table 1) which were fully inserted in all subjects. The frequency allocation of the electrodes is listed in S2 Table. All subjects had at least 1 year of experience in hearing with the CI (range from 1 to 6 years) and a monosyllabic speech perception score (Freiburg monosyllables [30]) of at least 50% on the implanted ear. For the bilateral EAS subjects the date of the first implantation was used to calculate the EAS experience. All subjects used a DUET2 processor (omnidirectional microphone sensitivity). Study tests were conducted with their daily used program without changing parameters. The hearing device on the contralateral ear remained unchanged, since all subjects confirmed sufficient hearing performance. Therefore, the documentation of the hearing performance with the hearing aid and the provided gain was not included in the study protocol.

All subjects gave their written consent for participation in this study. The study was approved by the local institutional review board (Ethical Review board of Faculty of Medicine, Goethe-University Frankfurt: No. 213/16, No. 394/12 for the EAS subjects). The tests were conducted in accordance with the Code of Ethics of the World Medical Association (Declaration of Helsinki) for experiments with humans. All participants were offered breaks from time to time to achieve consistent results.

### Sound localization setup

The mean localization error (MLE) in the horizontal plane was determined in a free field setup (anechoic chamber). The setup used was firstly described by Weißgerber et al. in 2014 [31]. A multi-channel setup with 128 loudspeakers (diameter: 3”) was used. Angles of sound presentation were between ± 60° (-2°, ± 21°, ± 42°, ± 59°) in the frontal and dorsal directions. The NH group was not tested for dorsal sound presentation since no significant difference in localization accuracy for frontal or dorsal sound presentation was found in a pilot study with NH subjects. Each direction was tested five times, so each run included 70 stimulus presentations for the EAS group and 35 presentations for the NH, respectively. The subjects were asked to turn their heads to the midline and to visually fixate an initial active LED at 0° prior to sound presentation to avoid additional cues from head movements [32]. The perceived direction was recorded using a pointing method adapted from the method proposed by Seeber et al. [33]. Instead of a laser pointer, a LED bar was positioned in the horizontal plane in front of the subject. The subject could display the perceived location of the signal by changing the active LED with a rotary encoder. The minimum change in angle between two LED positions was lower than 1° and the chance level to choose the closest LED was 0.9%. The frontal or dorsal direction could be selected with a toggle switch. The selected direction was displayed to the subject by changing the color of the active LED. A red LED color was coded for frontal sound presentation and a green LED color was used for the indication of dorsal sound stimuli. All subjects had an initial training phase with one presentation per tested loudspeaker. Therefore, the EAS users had 14 test stimuli and the NH had 7 test stimuli for training. In the training phase the supervisor was within the anechoic chamber to help the subjects. In the test phase the subjects were alone and supervised via webcam. No feedback was given to participants during the test procedure. The study tests were conducted in complete darkness to avoid the use of visual cues. Additionally, the loudspeakers were hidden behind an acoustic transparent curtain.

The stimuli were five noise bursts with a burst length of 30 ms (3 ms ramping) followed by a pause of 70 ms. Three different noise signals were tested for the EAS group: a low-pass (LP) noise (cut-off frequency 0.5 kHz, 48 dB/octave, butterworth filter), a high-pass (HP) noise (cut-off frequency 1.5 kHz, 48 dB/octave, butterworth filter) and a broadband (BB) white noise. The NH group was only tested with the broadband noise. The stimuli were presented at a level of 65 dB SPL with randomized level roving of ± 6 dB in steps of 3 dB to avoid use of monaural intensity cues. S1 Fig depicts temporal and spectral characteristics of the stimuli. The measurement setup, sound presentation and recording of the patient’s response were realized with the software MATLAB R2010a (MathWorks, Natick, MA, United States).

For each test angle the localization error was calculated as the difference between perceived and presented angle averaged (median) over the five measurements. The frontal and dorsal MLE was calculated as the localization error averaged over all frontal or dorsal test angles.

### JND-ITD setup

JND-ITDs were measured using a two-alternative forced choice method (2-AFC) with a 1 up-2 down procedure to estimate the threshold of 70.7% correct [34]. The test was carried out with pure tones of three different frequencies (125 Hz, 250 Hz and 500 Hz) with a length of 400 ms and a cosine ramp of 10 ms.

The stimuli were presented dichotically via headphones (Sennheiser HDA 200, Wedemark, Germany) to residual acoustic hearing without additional hearing supplies. An USB audio interface RME Fireface UC (Audio AG, Haimhausen, Germany) was used for digital to analog conversion and as a headphone amplifier. In this setup also the software MATLAB R2010a (MathWorks, Natick, MA, United States) was used to generate and present the sound stimuli and to record the patient’s response. The sound pressure level was 65 dB SPL for the NH group. In the EAS group, interaural loudness matching to consolidate symmetrical loudness perception on both sides was performed for each test frequency prior to the experiment. For this purpose, pure tone pulses were first presented to the one ear with better low-frequency hearing and the test person was asked to adjust a comfortable loudness level. Afterwards, subjects were asked to adjust the loudness of the other (poorer hearing) ear to elicit the binaural impression of a centered stimulus location inside the head. This procedure was repeated five times and the mean sound pressure level was chosen as presentation level for the stimuli in the study. This was done to achieve an ILD of zero for the ITD experiment. The stimulation levels are listed in S2 Table.

Two consecutive sound signals with opposing ITDs on each side and a pause of 400 ms between the signals were presented. The subjects were asked to indicate the change in the perceived sound position by choosing between two arrows pointing left or right on a graphical user interface on a tablet. The order of the signals with opposing ITDs was randomized. The method was adapted from earlier works by Gifford et al [26]. For training, the subjects completed the lateralization task in a run with ten presentations of a constant ITD of 500 μs. The EAS group completed the training for all frequencies, whereas the NH group performed the lateralization task only once with a random selected stimulus frequency. If the training runs were successfully completed, the starting value of the ITD in the first experimental run was set to 500 μs. If the subject was unable to correctly indicate the direction of the signal in the training phase, the starting value was increased. The initial step size was 100 μs and halved after two reversals with a minimum step size of 25 μs. The run was completed after six reversals with a minimum increment and was forced to terminate after 80 iterations. The JND was calculated using the so-called “mid estimation run”, which is described by the mean of the last six extremes [34].

The EAS group repeated the test three times for each frequency. The NH group performed one iteration per frequency. The JND-ITDs in the EAS group were calculated as the mean of the three trials for each frequency. The order of the frequencies was randomly chosen to avoid any bias caused by training effects [5].

### Statistics

For descriptive statistics, boxplots and the description of median values were used throughout the manuscript. Nonparametric tests were used for statistical analysis of MLE and ITD sensitivity. For pairwise comparison of subject groups, the Mann-Whitney-U-test was used to compare the results of the EAS cohort with those of the NH group. The Wilcoxon signed rank test with adjustment of alpha (Bonferroni correction) was used for tests of paired samples within the subject groups. The tests comparing more than two related samples were performed using Friedman’s test (one-way repeated measure analysis of variance by ranks). Correlations were tested via Spearman rank correlation. A p-value < 0.050 was considered significant. IBM SPSS Statistics 24 (IBM, Armonik, New York) was used for the analysis.

## Results

### Sound localization accuracy

**Fig 2:**
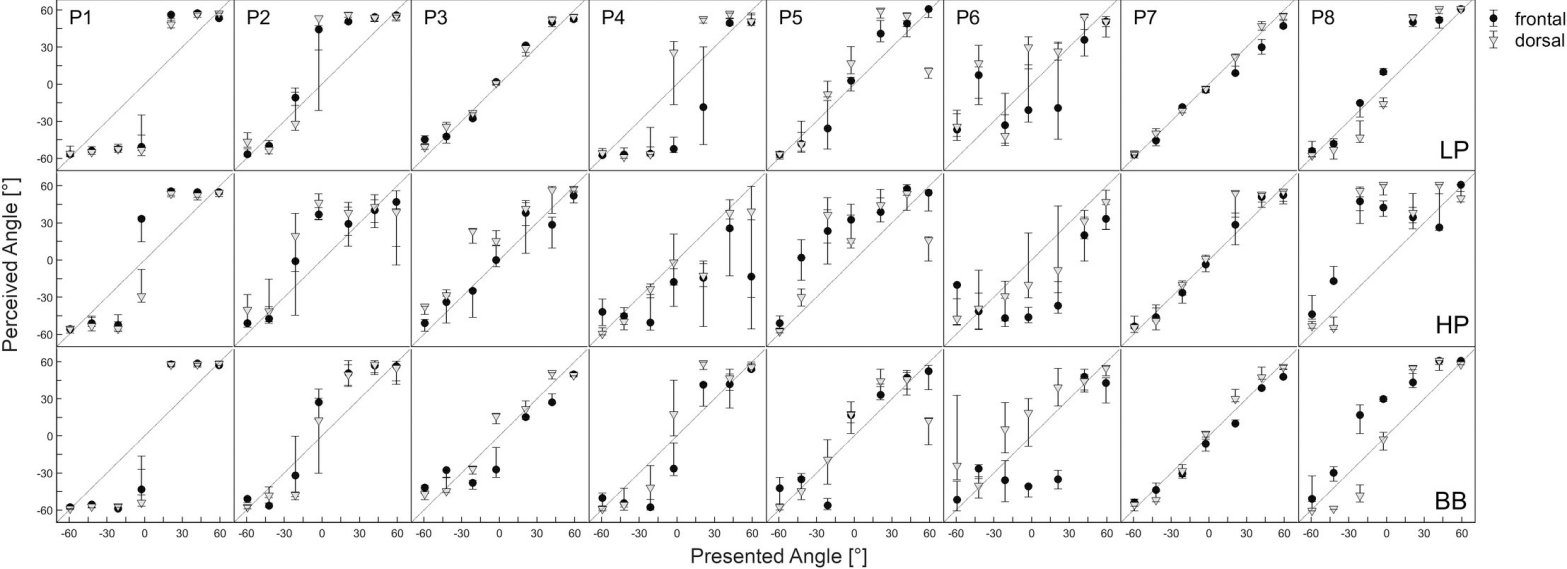
Individual localization accuracies for 8 EAS subjects depending on stimulus spectral characteristics. LP: low-pass filtered noise, HP: high-pass filtered noise, BB: broadband noise. Dots: frontal sound incidence, triangles: dorsal sound incidence. Whiskers: 1^st^ and 3^rd^ quartile. Diagonal: perfect localization (perceived angle equal to presented angle). P1+P7 bilateral EAS, P2- P6 bimodal support (with hearing aid on opposite side), P8 no hearing aid on opposite side.

The individual accuracy of localization for the EAS cohort for the three bandpass stimuli are depicted in Fig 2. All subjects were somehow able to discriminate different directions of sound incidence direction. The EAS group had difficulties in distinguishing the frontal and the dorsal directions. The median amount of front/back confusions was 47% for the low-pass noise, 47% for the high-pass noise and 46% for the broadband stimulus and was thus, on chance level. In the EAS group no significant difference in MLE was found between the frontal and the dorsal presentation for all stimuli (*low-pass*: *z = -1*.*752*, *p = 0*.*08; high-pass*: *z = -0*.*420*, *p = 0*.*674; broadband*: *z = -0*.*700*, *p = 0*.*484*). Therefore, further analysis were only conducted for the frontal condition. The MLE of the individual NH subjects is found in S1 Table and of the EAS subjects is in S2 Table.

**Fig 3:**
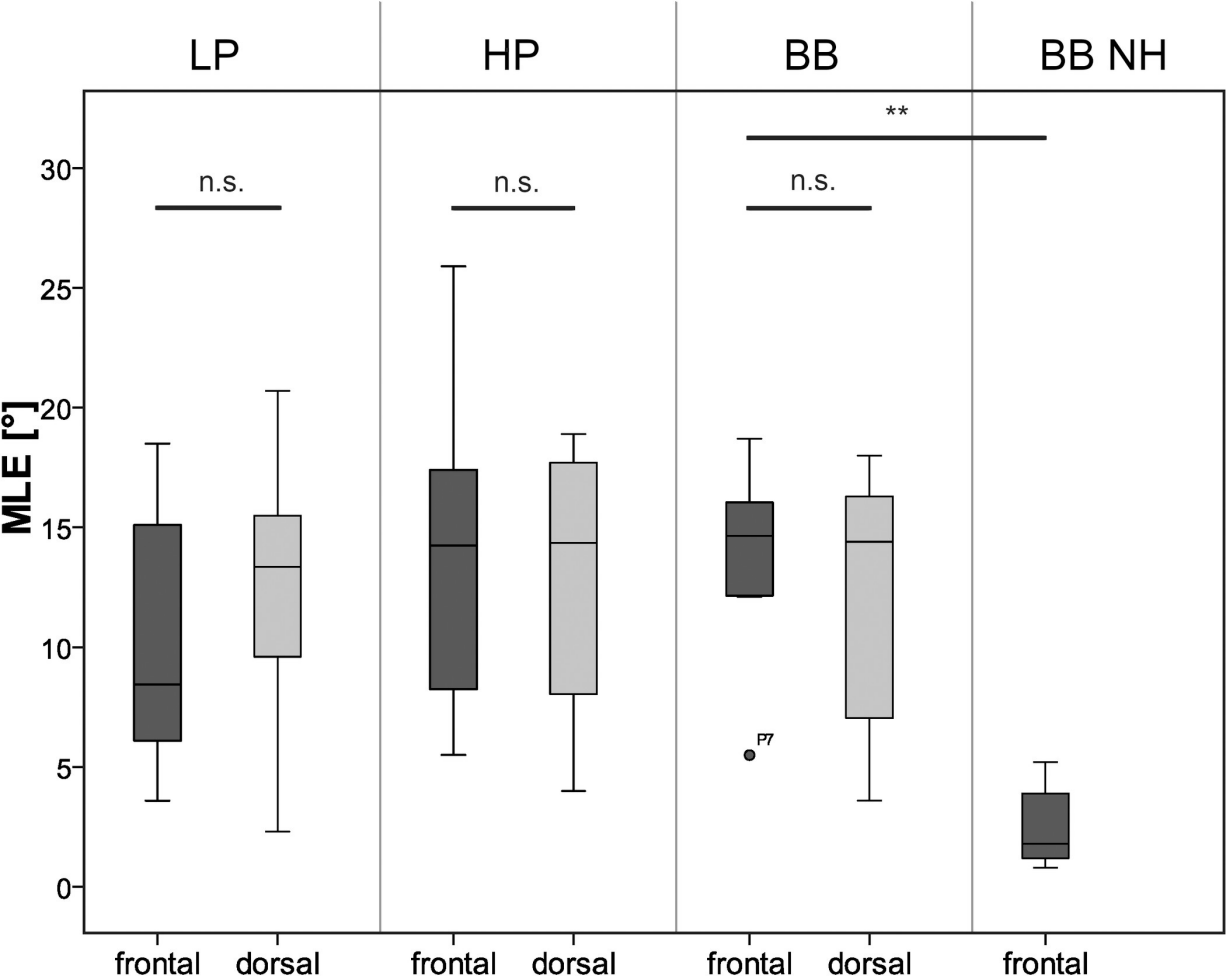
Localization error (MLE) for low-pass (LP), high-pass (HP) and broadband (BB) filtered noise, EAS cohort (n = 8). BB NH: MLE broadband signals of NH subjects (n = 24). Sound incidence either frontal (dark) or dorsal (grey). Boxplots contain median, 1^st^ and 3^rd^ quartiles and the minimum and maximum values. Circles indicate outliers.

The MLE (frontal) for broadband stimuli was 1.8° for the NH group. In the EAS group an average MLE of 8.5° was found for low-pass signals, 14.3° for high-pass signals and 14.7° for broadband signals, respectively. Boxplots of the MLE are shown for both groups in Fig 3. The NH group achieved a significantly lower average MLE for broadband stimuli than the EAS group (*z = -4*.*186*, *p < 0*.*001*). In the EAS group, there was no significant impact of the spectral characteristics of the test stimuli on MLE (*df = 2*, *χ = 3*.*161*, *p = 0*.*206*).

### JND-ITD

**Fig 4:**
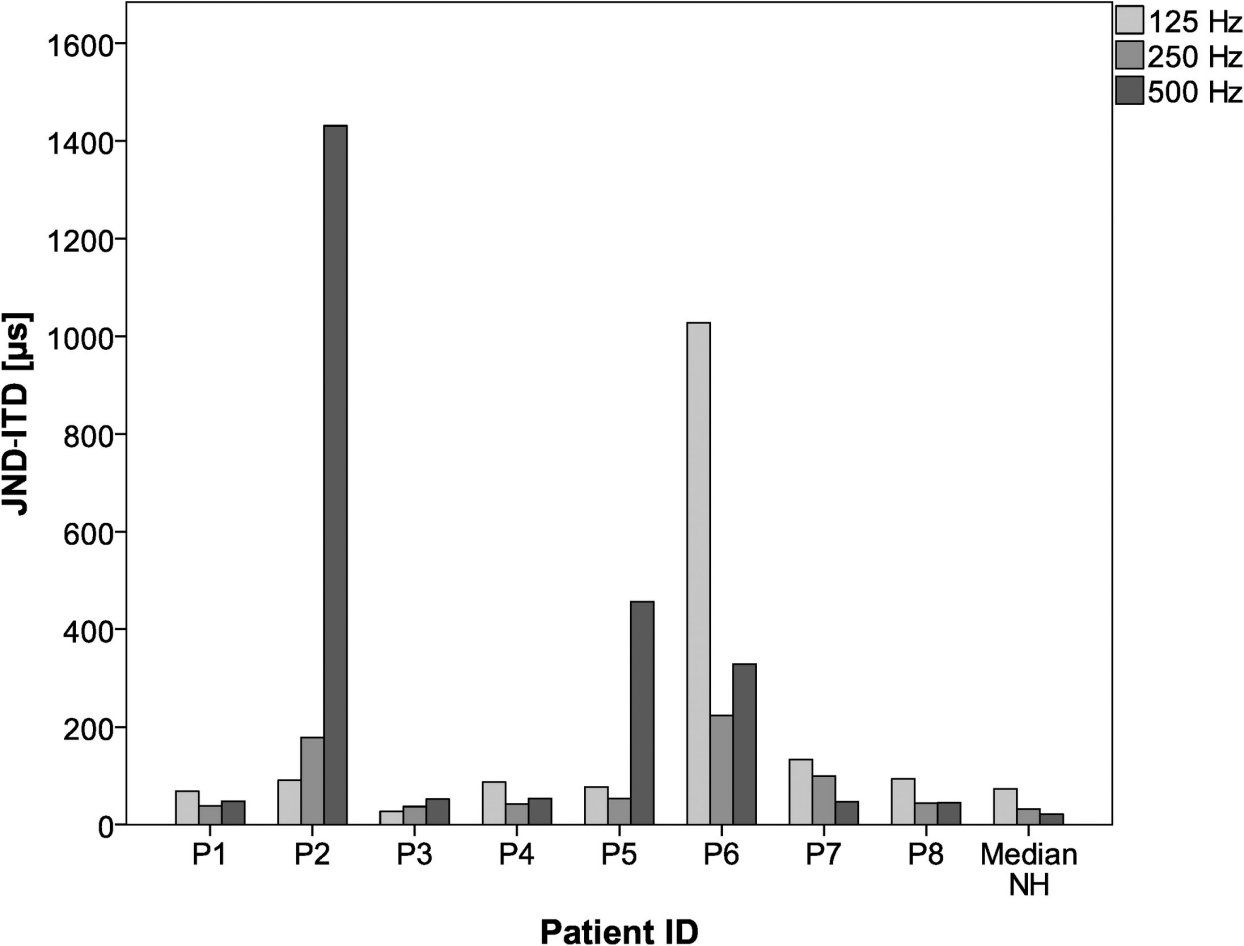
Mean individual JND-ITDs (EAS subjects) and NH median results (n = 24) depending on test stimulus frequency (sinusoids).

Individual JND-ITD for the EAS subjects as well as median JND-ITD for the NH are depicted in Fig 4. Median JND-ITD for the EAS subjects was 88.7 μs for 125 Hz, 48.8 μs for 250 Hz and 52.9 μs for 500 Hz, respectively. For 125 Hz and for 500 Hz, two EAS subjects showed results beyond the physiological limit of around 700 μs, which corresponds to a sound incidence direction of 90° [27]. Both subjects improved ITD sensitivity over the three repetitions. The JNDs of the NH group were smaller for all stimuli with a median of 73.3 μs for 125 Hz, 32.1 μs for 250 Hz and 21.7 μs for 500 Hz. The JND-ITDs of the NH participants is in S1 Table and the results of the EAS subjects are found in S2 Table.

**Fig 5:**
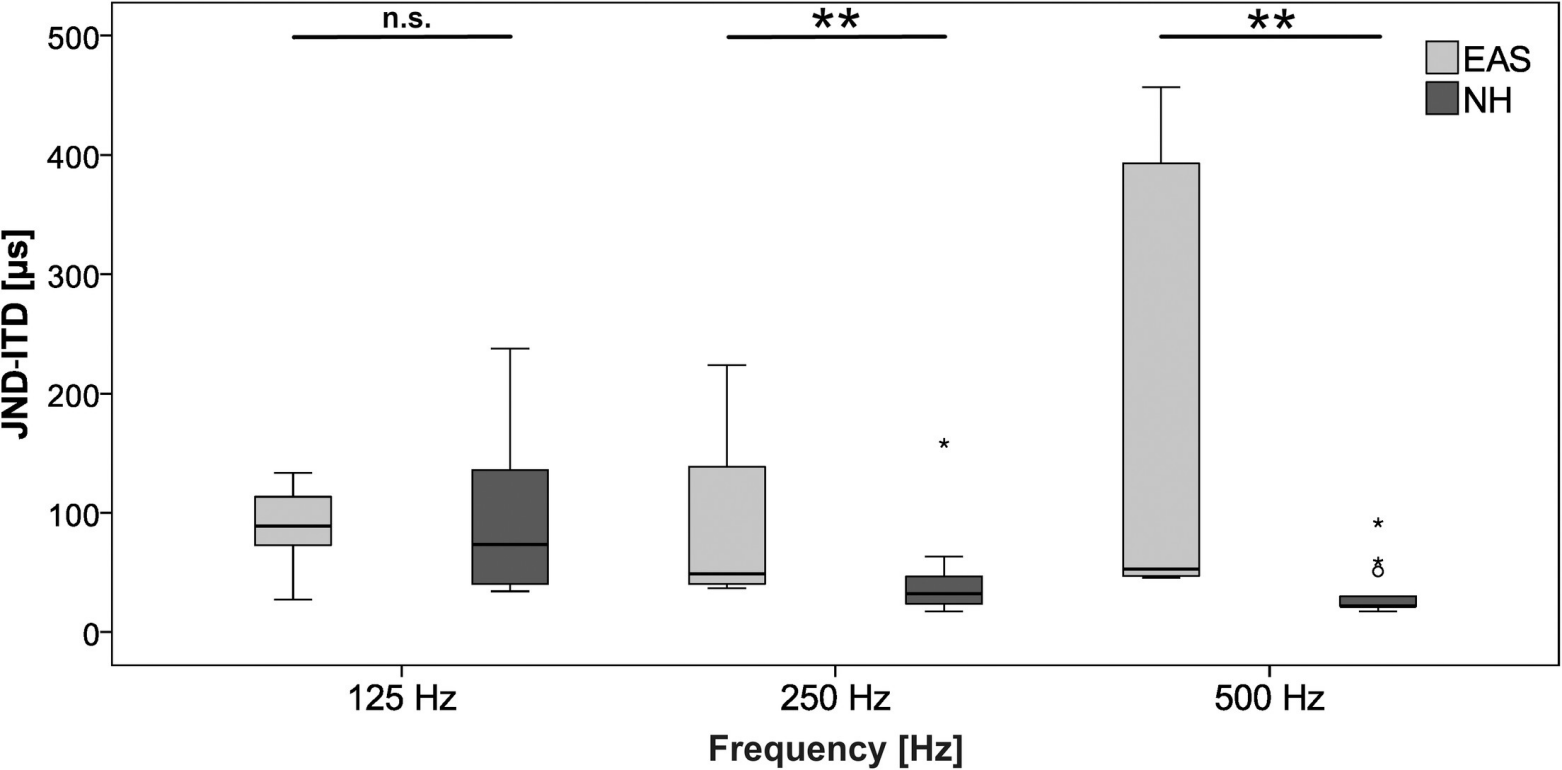
Boxplots of JND-ITDs of EAS (n = 8) and NH (n = 24) group depending on test stimulus frequency. Two outlier data points not indicated (P6: 125 Hz, 1027.8 μs; P2: 500 Hz, 1431 μs). Stars at boxplot indicate extreme outliers.

In the EAS group, there was no impact of test stimulus frequency on JND (*df = 2*, *χ = 4*.*750*, *p = 0*.*093*). In the NH group, the JND-ITDs differed significantly depending on test stimulus frequency (*df = 2*, *χ = 29*.*872*, *p < 0*.*001*). A pairwise comparison revealed a significant difference in JND-ITDs for 125 Hz compared with the other two test frequencies, but no significant difference between the JND-ITDs of 250 Hz and 500 Hz was found (*125 Hz to 250 Hz*: *z = -3*.*944*, *p < 0*.*001; 125 Hz to 500 Hz*: *z = -4*.*258*, *p < 0*.*001 and 250 Hz to 500 Hz*: *z = -1*.*706*, *p = 0*.*088*). Boxplots of both groups for the different test frequencies are plotted in Fig 5. No significant difference between subject group was found for the test frequency 125 Hz (*z = -0*.*610*, *p = 0*.*564*). The results of the JND-ITD at 250 Hz and 500 Hz differed significantly between subject groups (*250 Hz*: *z = -2*.*552*, *p = 0*.*009; 500 Hz*: *z = -3*.*457*, *p < 0*.*001*).

The interaural asymmetry of pure tone audiogram thresholds at 500 Hz correlated significantly with the JND-ITD of 500 Hz for the EAS subjects (*r*_*s*_ *= 0*.*85*, *p = 0*.*007*), whereas there were no significant correlations between interaural asymmetry and the JND-ITDs for 125 Hz and 250 Hz (*125 Hz*: *r*_*s*_ *= 0*.*259 p = 0*.*535*, *250 Hz*: *r*_*s*_ *= -0*.*368*, *p = 0*.*369*).

A significant correlation was found between the mean of pure tone audiogram thresholds and the JND-ITDs for 125 Hz and for 250 Hz but not for 500 Hz (*125 Hz*: *r*_*s*_ *= 0*.*731*, *p = 0*.*040*, *250 Hz*: *r*_*s*_ *= 0*.*724*, *p = 0*.*042*, *500 Hz*: *r*_*s*_ *= 0*.*467*, *p = 0*.*467*).

The NH group was significantly younger than the EAS group (*z = -3*.*856*, *p < 0*.*001*). No age effects were found in nearly all MLE and JND-ITD results (one exception: NH: JND-ITD of 500 Hz and age: *r*_*s*_ *= 0*.*455*, *p = 0*.*026)*.

No correlations were found in the EAS group between the speech perception (Freiburg monosyllable speech test) or the EAS experience and the measured MLE (LP, HP, BB noise) and the JND-ITDs (125 Hz, 250 Hz, 500 Hz respectively). The MLE of LP signals did not correlate significantly with the JND-ITDs (125 Hz, 250 Hz and 500 Hz).

## Discussion

Eight CI users with EAS (6 unilaterally implanted, 2 bilaterally implanted) and 24 NH subjects underwent evaluation of MLE (low-, high-pass and broadband noise stimuli) and ITD sensitivity (125 Hz, 250 Hz and 500 Hz pure tone stimuli). All EAS subjects were able to localize sounds. However, the EAS group showed decreased localization accuracy compared to the NH group. The MLE range for broadband signals for EAS users varied from 5.5° to 18.7° (NH range: 0.8° to 5.2°). The EAS user with the best performance achieved a MLE of 3.6° for the low-pass signals, reaching the range of subjects with normal hearing. EAS users could not distinguish between a frontal and a dorsal sound presentation with a mean amount of front/back confusions at chance level. EAS users were able to detect ITDs in different low-frequency sinusoids (125 Hz, 250 Hz and 500 Hz). ITD sensitivity was well below the physiological threshold of around 700 μs, with the exception of two subjects who reached JND-ITDs above this threshold at two different test frequencies (P2: 1,431 μs at 500 Hz, P6: 1,027.8 μs at 125 Hz).

The MLE of low-pass stimuli for EAS users is slightly (not significant) better compared to high-pass and broadband stimuli. But the assumption of improved localization accuracy (reflected by smaller MLE) for low-pass signals compared to high-pass signals could not be confirmed, although they are sensitive to acoustic ITDs in the lower frequencies. ITD sensitivity was not significantly different for 125 Hz between the EAS cohort and the NH group, where the interaural asymmetry of pure tone thresholds was the slightest and the pure tone audiogram thresholds were the lowest.

### Sound localization accuracy

Comparing different groups of CI users, EAS users demonstrated nearly equal sound localization accuracy for broadband stimuli compared to a cohort of patients with single-sided deafness (13.1° in aided condition, tested in the same laboratory setup) [35]. Bimodal CI users (using a contralateral hearing aid with CI without EAS in the ipsilateral ear) achieved a poorer MLE of around 20° [31]. The localization accuracy of the NH for broadband stimuli was in good agreement with the results of NH by Seeber with a comparable localization setup (1.8° in our study and 1.6° in Seeber’s study) [33]. Localization accuracy of the EAS subjects as reflected by MLE obtained with low-pass, high-pass and broadband noise stimuli did not differ. This is partly in line with earlier results reported by Loiselle et al. [36], as they found no significant difference in MLE for low-pass and broadband signals in a group of EAS subjects. However, a significant decrease in localization accuracy for high-pass signals was found there, whereas in our study no significant difference was found for MLE of low-pass, high-pass and broadband stimuli.

A comparison of study results on sound localization accuracy is only possible to a certain extent, since differences in methodological issues have an effect on the study outcome. The experimental setup in Gifford et al. [26] comprised a loudspeaker span of ± 90°, whereas in the present study a span of ± 60° degrees was used to assess the MLE. It is know that localization error reaches its maximum at 90° [1]. Therefore, a higher MLE is expected for a loudspeaker span of ± 90°. Consequently, a difference in root mean square localization error for broadband signals in EAS subjects between Gifford et al. (43.4°) and the present study (18.6°, MLE calculated as root mean square error) was found.

Furthermore, differences in the angular resolution of response recording lead to differences in chance level. In the study of Gifford and collaborators [26] nine loudspeakers were used for sound presentation and 24 dummy loudspeakers were used to improve the angular resolution of response recording (5.5°, chance level: 3%). In the study of Loiselle and colleagues [36] the resolution was 15° (chance level: 8%). In the present study the resolution of response recording was less than 1°. The finer resolution of response recording could be a potential factor to affect MLE results of similar studies. Another aspect is the mostly visible arrangement of loudspeakers which allows the subject for a discretization of possible angles of sound incidence, whereas in the present configuration the loudspeakers were covered by an acoustically transparent curtain to minimize visual cues.

The assessment of sound localization accuracy may also depend on characteristics of the stimulus. The present setup was resembling the procedure described by Seeber [33], and applied the same stimulus (multiple short noise bursts), while the studies mentioned above used single noise bursts of 200 ms duration. In the present study multiple signal onsets potentially provide more apparent cues to ameliorate localization accuracy. In particular, signal onsets are analyzed to obtain localization cues [37, 38]. A recent study compared localization accuracy depending on stimulus characteristics in NH [39]. The authors reported that pulsed stimuli lead to more accurate localization compared to continuous stimuli. This might explain in addition to other methodological differences the here reported more accurate localization results compared to other studies.

Finally, differences in study subjects presumably could explain diverging results. The minimum EAS experience of test subjects reported by Gifford and colleagues was 6 months with an average experience of 3.5 years, whereas the minimum experience in the present study was 12 months. However, mean EAS experience of 3.6 years was similar.

### JND-ITD

The JND-ITDs for the low-frequency signal (125 Hz) in EAS users and in the NH control group are at a comparable level. One EAS user showed a remarkable JND-ITD of only 27.2 μs at 125 Hz. The present results indicate that ITD sensitivity for lower frequencies is not affected in the EAS users studied here. This finding is potentially the consequence of near to normal hearing thresholds and therefore small or absent interaural asymmetry in the lower frequency range. Correspondingly significant correlations were found between the mean pure tone audiogram thresholds and the JND-ITDs for 125 Hz and 250 Hz in the EAS subjects. For the stimulus frequency of 500 Hz, at which residual hearing is worse and interaural asymmetry is larger, the JND-ITDs correlated significantly with interaural asymmetry of pure tone audiogram thresholds (but not mean pure tone audiogram thresholds). The larger interaural asymmetry is potentially the reason for the significant difference in JND-ITD between the EAS cohort and the NH group for higher test frequencies. A dependence of binaural cue sensitivity on a small interaural asymmetry of the pure tone thresholds was also reported by Loiselle et al. [28]. In their study, subjects with symmetric residual hearing showed better localization accuracy compared to subjects with asymmetric hearing loss. They compared the localization accuracy of eight bimodal EAS subjects (with contralateral hearing aid) with low interaural asymmetry (below 15 dB at 250 Hz) with four bimodal EAS subjects with larger interaural asymmetry and found a significant decrease in localization performance for the cohort with asymmetrical pure tone thresholds. Gifford et al. [26] also reported significant correlations between interaural asymmetry in hearing loss and the JND-ITD for low-pass noise stimuli, as well as for a sinusoid of 250 Hz. One difference in the studies was that the mean interaural asymmetry in pure tone audiograms of the present study was lower (7.5/ 11.9/ 16.3 dB HL at 125 Hz, 250 Hz, and 500 Hz), whereas Gifford et al. reported larger asymmetries at the low frequencies (14.6, 16.8 and 22.1 dB HL). In addition, the ITD thresholds reported by Gifford et al. were in a wider range between 43 μs and over 1600 μs (two subjects unable to track ITD cues at all). Potentially, the wider range of interaural asymmetry and JND-ITDs led to a higher correlation.

On the other hand, it was assumed that the position of the electrode inside the scala tympani potentially increases the stiffness of the basilar membrane, and therefore distorts the transmission of the traveling wave. This study showed that EAS subjects can perceive very small JND-ITDs below 100 μs at lower frequencies. Thus, it can be assumed that an undistorted acoustic low-frequency hearing with precise timing in the cochlear is possible even with an inserted electrode array.

## General discussion

No significant correlation was found between MLE and ITD sensitivity for any of the frequencies tested in the JND-ITD setup. Similar to previous studies, it was assumed that the ability to detect ITDs leads to improved localization accuracy, especially for low-pass filtered signals [28].

It has to be considered that the cohort of EAS users of the present study was rather small. In addition, the device delivering acoustic amplification was varying between subjects (2x EAS-CI processor [bilateral users], 5x hearing aid, 1x open ear). This heterogeneity might disguise potential correlations between accuracy of localization and JND-ITDs, since accuracy was measured with different modes of amplification, while JND-ITD was determined with headphone presentation of stimuli. The delay introduced by signal processing in the hearing aid (on average between 3 ms and 11 ms [11]) potentially distorts the transfer of temporal binaural timing cues [40].

Although Yost reported in 2017 that envelope ITD plays a minor role in acoustic localization accuracy in NH [2], sensitivity to envelope ITDs could have improved the localization accuracy across stimuli in our EAS cohort. Moore and colleagues assumed that the sensitivity to envelope ITDs and temporal fine structure ITDs is processed partially independently in the auditory system of NH [41]. They reported ITD thresholds conveyed only by envelope or temporal fine structure cues and a combination of both. ITD thresholds improved even with uninformative envelope cues. In addition, high individual ITD threshold variability was observed with contradicting lateralization cues. This confirmed the assumption of an at least to some extent independent processing of both lateralization mechanisms. Further studies on the relevance of envelope ITDs and temporal fine structure ITDs in EAS users must be carried out in order to receive insights into binaural localization cue sensitivity in this special patient cohort.

Since average age differed between EAS and NH groups in the present study, a potential impact of age on acoustic localization performance needs to be discussed. Several studies reported a significant age effect on ITD sensitivity, determined via psychoacoustic [41, 42] or objective measurements [43–45]. However, in the present study a significant effect of age on accuracy of localization (MLE) or sensitivity to interaural time differences (JND-ITD) was absent in both the EAS and NH cohort (except for a significant correlation between JND-ITD 500 Hz and age within the NH group). Also no significant age effect was reported for ILD and ITD sensitivity for young and early elderly in another study. The performances of the ITD and ILD tasks were not significantly different between the tested age groups [46].

Subjects using bilateral EAS are still rare. Only two bilateral EAS patients could be recruited in the present study. These two bilateral EAS participants showed very diverging results. One of the subjects (P7) achieved the best localization accuracy within the EAS group (MLE of 5.5° for broadband stimuli, JND-ITD of 125 Hz: 133 μs, 250 Hz: 99 μs and 500 Hz: 47 μs). However, the other participant (P1) demonstrated poorer MLE (15° for broadband stimuli) but better JND-ITDs (125 Hz: 69 μs, 250 Hz: 38 μs and 500 Hz: 48 μs). The considerable discrepancies in localization accuracy could be associated with the difference in the development of hearing impairment in these two study participants (P7: normal hearing for 36 years, P1: hearing impairment since birth, CI implantation as adult). It can be assumed that early hearing loss prevents the development of accurate processing of complex binaural information, caused by insufficient transfer of binaural cues. Furthermore, the delay between the first and second ear CI provision is potentially confounding (P7: 1.5 y, P1: 5 y) as it was shown in children that a longer inter-implant interval decreased localization accuracy [47]. A normal binaural hearing development seems to be crucial for obtaining access to the relevant binaural cues, but additional tests with subjects with this hearing supply are necessary to conclude potential hearing effects.

## Conclusion

CI EAS users with sufficient bilateral hearing in the low-frequency range are able to detect acoustic ITDs with thresholds close to normal hearing. In an acoustic localization task, however, the accuracy of normal hearing was not achieved and the detection of front/back sound incidence was on chance level. The transmission of accurate binaural time information by the devices used for acoustic amplification on both sides should be ensured in the future.

## Supporting information

S1 Table Demographic data and test results of NH subjects. (XLSX)

S2 Table Demographic data and test results of EAS subjects. (XLSX)

S1 Fig Sound localization stimuli in temporal (upper) and frequency domain (lower). Stimuli of the frontal loudspeaker (located at -2°) for the low-pass (1^st^ row), high-pass (2^nd^ row) and broadband (3^rd^ row) filtered signals. Stimuli include compensation of individual loudspeaker frequency response. Plots of frequency responses were smoothed for better readability.
(TIF)
