# Supplementary figures and images for: Sensitivity to interaural time differences and localization accuracy in cochlear implant users with combined electric-acoustic stimulation
Source: PLoS One. 2020 Oct 19;15(10):e0241015. doi: 10.1371/journal.pone.0241015 (PMC7571672; doi:10.1371/journal.pone.0241015)

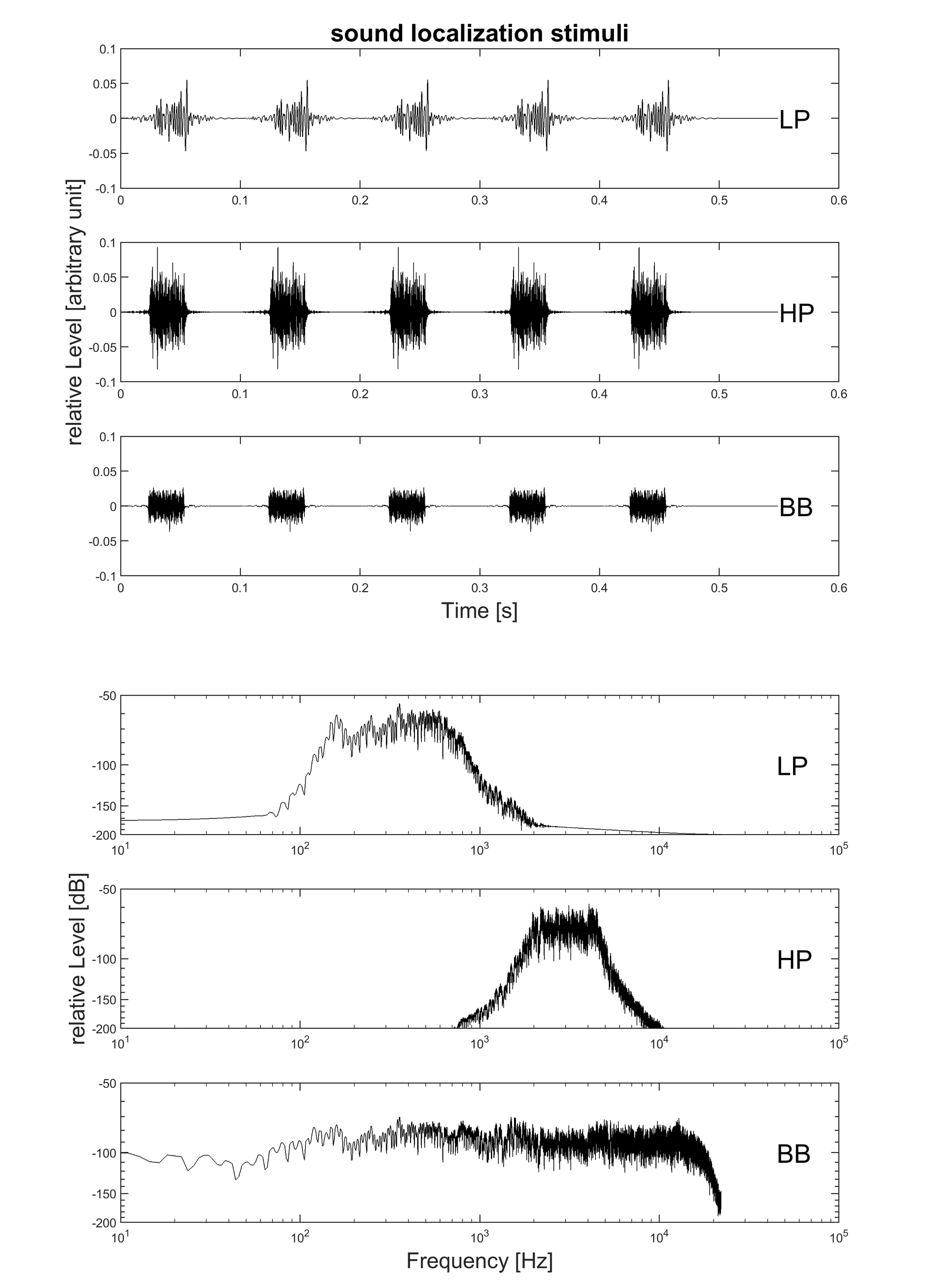

Supplement: S1 Fig — Stimuli of the frontal loudspeaker (located at -2°) for the low-pass (1st row), high-pass (2nd row) and broadband (3rd row) filtered signals. Stimuli include compensation of individual loudspeaker frequency response. Plots of frequency responses were smoothed for better readability. (TIF) [file pone.0241015.s003.tif]
